# Allele-specific expression elucidates *cis*-regulatory logic

**DOI:** 10.1371/journal.pgen.1007690

**Published:** 2018-11-01

**Authors:** Sofie Y. N. Delbare, Andrew G. Clark

**Affiliations:** Molecular Biology and Genetics, Cornell University, Ithaca, New York, United States of America; New York University, UNITED STATES

Most people who tune pianos do not have perfect pitch. In the days before electronic tuners, they would accomplish this feat with amazing precision by listening for the rate at which two notes, struck simultaneously, would “beat” against each other. The contrast of two tones can allow interval tuning with a pitch error well under 0.1%. Throughout the sciences, methods that contrast two signals have provided massive improvements in precision. The interferometer in its various forms has allowed comparison of two signals to achieve precise measurement of the speed of light, the existence of gravitational waves, and quantum entanglement. In functional genomics, one of the most precise ways to compare gene expression levels is to contrast the expression of the two alleles in heterozygotes using an approach called allele-specific expression (ASE). By quantifying the relative counts of a cell’s transcripts from two alleles that differ by single nucleotide polymorphisms (SNPs), ASE has been used to contrast *cis*- versus *trans*-regulation of genes [1] and to examine genomic imprinting [2] and escape from X chromosome inactivation [3]. In this issue, we see another excellent application of this approach to explore differences in spatial patterns of expression in the early *Drosophila* embryo.

## Advances in expression profiling in the *Drosophila* embryo

The *Drosophila* embryo provides a classic model for pattern formation during development. Much progress has been made in mapping the spatial and temporal gene regulatory circuits that pattern cell fate in the embryo. Spatial gradients of gene expression are strictly controlled along the anterior-posterior and dorso-ventral axes. This is mediated by *cis*-regulatory modules (CRMs) that respond to maternal and zygotic *trans*-acting factors that are differentially distributed along the axes [4]. Early methods of analysis relied on beautiful immunofluorescence-tagged images of fly embryos, probing one or a few genes at a time. Now, modern genomic approaches allow whole-genome gene expression measurements that enable construction of 3D gene expression maps of organisms, as done recently using single-cell RNA sequencing in late-stage fly embryos [5]. Similar attempts to enhance spatial resolution have been done by cell-specific barcoding and fluorescence activated cell sorting (FACS) of specific neurons in *Caenorhabditis elegans* [6] and 2D-arrayed bar-coded primers for RNA sequencing of tissue sections [7]. Concurrently, efforts have been done to more precisely delineate temporal transcriptional differences in the maternal-zygotic transition in *Drosophila* [8] and across tissues [9] and developmental stages in *C*. *elegans* [10]. Because these organisms have two copies of the genome, CRMs can differ between alleles of each copy. Thus, quantification of ASE can augment these studies by allowing us to ask which sequences on the same allele (in *cis*) are important in orchestrating gene expression during development.

## Spatially resolved ASE in F1 hybrid embryos

**Fig 1:**
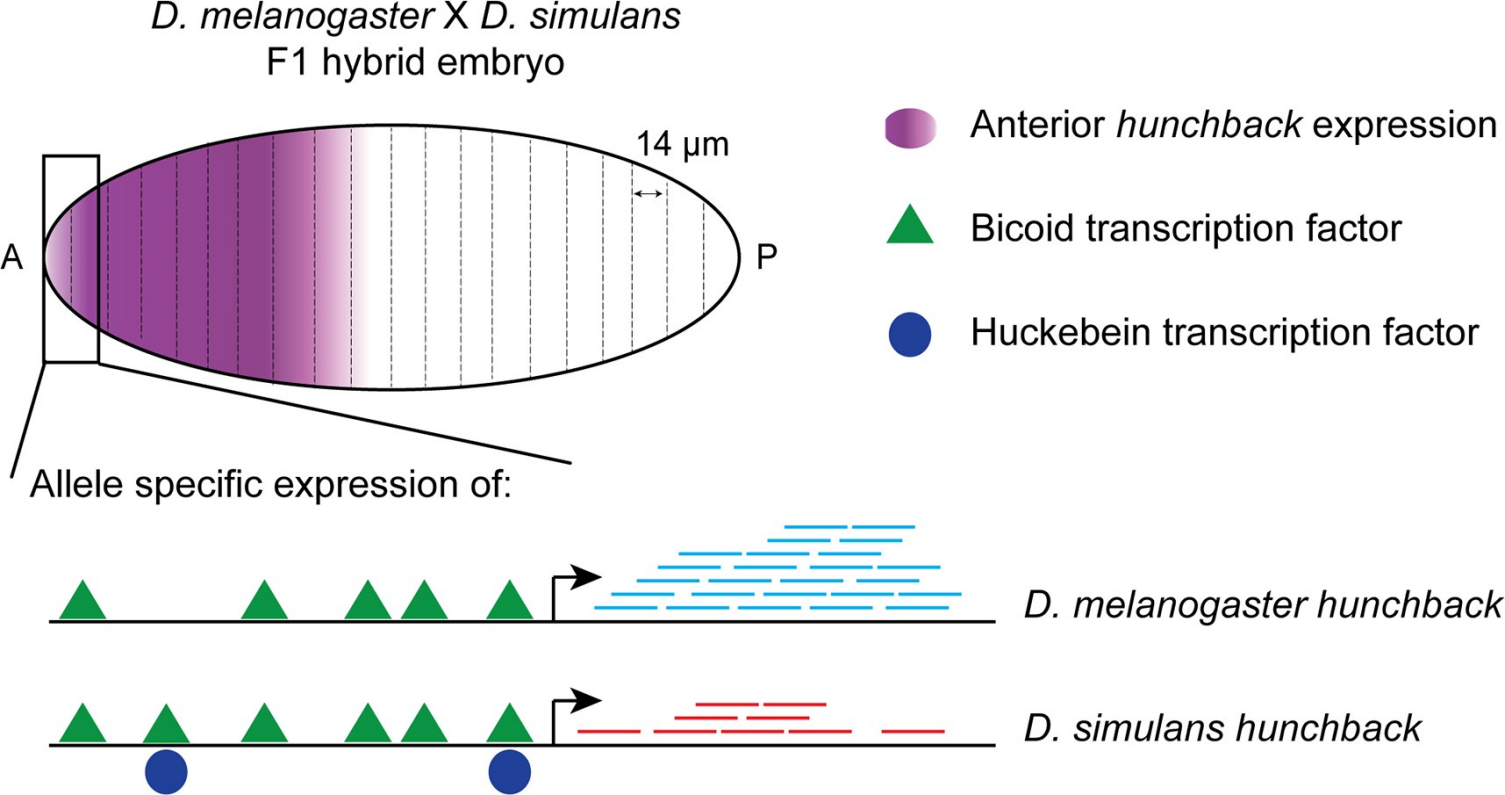
Schematic of Combs and Fraser's model for ASE of *hunchback*. In the F1 hybrid embryo (from a *D*. *melanogaster* × *D*. *simulans* cross), there is a maternal gradient of Bicoid protein, with greater concentration toward the anterior. Bicoid acts as a *trans*-activator of the zygotic gene *hunchback*, which has CRMs upstream of the transcription start-site (arrow). The canonical CRM has a cluster of binding sites for Bicoid, indicated by green triangles. The *melanogaster* and *simulans* alleles of *hunchback* differ in that the *simulans* form has an additional (sixth) Bicoid binding site in its canonical CRM, and it has two weak binding sites for the transcription factor Huckebein, which could act as a repressor. Given these differences in their CRMs, the two species’ *hunchback* alleles “see” the *trans*-acting milieu differently. In F1 hybrid embryos, there is greater expression of the *melanogaster* allele at the anterior end (blue [*melanogaster*] versus red [*simulans*] transcripts; detected by identifying SNPs in the RNA sequencing reads). Further posterior in the embryo, Bicoid concentration is lower, and the two alleles of *hunchback* are transcribed at equal levels. ASE, allele-specific expression; CRM, *cis*-regulatory module; SNP, single nucleotide polymorphism.

Combs and Fraser [11] extend these methods by applying spatially resolved genome-wide ASE analysis to *Drosophila* embryos, to identify candidate genes involved in *cis*-regulatory divergence. Earlier studies often measured ASE in whole organisms, averaging transcription factor activity across tissues and cell types [12]. This can lead to an underestimation of ASE signals. To overcome this limitation, Combs and Fraser measured transcript profiles in 14-μm–thick cryo-sections that were made along the anterior-posterior axis of F1 hybrid embryos of *D*. *melanogaster* and its sister species *D*. *simulans* (Fig 1). This approach identified 66 genes whose patterns of ASE varied along the embryo. One of these genes, *hunchback*, encodes a transcription factor that is expressed anteriorly during *Drosophila* development and is required for formation of the fly’s anterior segments [13]. Anterior *hunchback* expression is higher in the *melanogaster* embryo compared to the *simulans* embryo, and this same *melanogaster* bias persists in ASE in the hybrid embryo, suggesting that the difference is *cis* based. Combs and Fraser then focused tightly on *hunchback*, seeking to identify binding site differences in the *melanogaster* and *simulans* CRMs that might explain the observed ASE bias.

## A single SNP drives species-specific spatial *hunchback* expression

Using a modeling approach [14], the authors narrowed down the transcription factor binding motifs in the *hunchback* CRMs with the largest effects on *hunchback* expression. The model singled out the canonical CRM (most proximal to the promoter), which has five Bicoid binding sites in *melanogaster*. In *simulans*, there is a sixth Bicoid binding site that also shows weak binding for the transcription factor Huckebein, which could act as a repressor. To functionally confirm that these differences in the canonical CRM are responsible for ASE of *hunchback*, Combs and Fraser edited the endogenous locus in *melanogaster* using CRISPR to have a *simulans*-like canonical CRM, with an extra Bicoid and Huckebein binding site. The edited line was crossed to a *simulans* line whose canonical CRM contains polymorphisms that make it more similar to *melanogaster*’s. In the resulting F1 hybrid, there was no longer an expression bias of the *melanogaster* allele when controlled by the *simulans* CRM, thus providing evidence that the *simulans* CRM reduces expression of whichever coding allele it controls. Whether this is directly due to the binding of Huckebein remains to be determined.

## Implications for the evolution of development

Because ASE is the read-out of differential *cis-* and *trans-*regulation, the molecular mechanisms that drive ASE can only be elucidated if comprehensive information regarding the presence of *cis*-regulatory regions, transcription factor binding motifs, and epigenetic modifications is known across tissues, cell types, timing in development, and disease states. Such work is ongoing in model organisms, using modeling in silico and using large-scale functional assays, including chromatin immunoprecipitation (ChIP) sequencing and fluorescent reporters [15, 16]. However, in the meantime, ASE in hybrids between model organisms and closely related sister species can help answer important questions related to the evolution of *cis*-regulation.

In dissecting one instance of species-specific spatially localized ASE, Combs and Fraser confirmed CRM divergence for *hunchback* that results in spatially distinct expression patterns between *melanogaster* and *simulans* embryos, despite the fact that these species are virtually indistinguishable in body plan. This observation is consistent with earlier studies that found abundant differences between *melanogaster* and *simulans* in both *cis*- and *trans*-regulation, despite the endpoint having minimal phenotypic divergence [17]. Across embryos of multiple fly species, mRNA levels are much more highly conserved than individual transcription factor binding events, consistent with the existence of compensatory *cis* and *trans* changes [18]. Such compensatory *cis* and/or *trans* variation might be neutral but could be selectively favored under certain conditions [19]. In this regard, ASE analysis is a powerful tool in genome-wide studies to dissect the adaptive nature of changes in gene regulation and how those changes impact development, behavior, and disease [20]. This new study adds a spatial dimension to the analysis of ASE and opens the door for many novel applications for dissecting *cis*- and *trans*-regulation of gene expression.
